# Blood Pressure Control and Anthropometric Differences in Afro-Descendants and Other Ethnic Groups in Hypertensive Brazilian Populations

**DOI:** 10.5334/gh.1448

**Published:** 2025-07-11

**Authors:** Maicon Borges Euzébio, Priscila Valverde de Oliveira Vitorino, Andréa Araújo Brandão, Eduardo Costa Duarte Barbosa, Audes Diógenes M. Feitosa, Marcus Vinícius Bolivar Malachias, Marco Mota Gomes, Celso Amodeo, Rui Manoel dos Santos Póvoa, Renato Delascio Lopes, Paulo César Brandão Veiga Jardim, Ana Luiza Lima Sousa, Ana Carolina Arantes, Antonio Coca, Weimar Kunz Sebba Barroso

**Affiliations:** 1 Unidade de Hipertensão Arterial da Universidade Federal de Goiás e Instituto Federal de Goiás, Brazil; 2 Pontifícia Universidade Católica de Goiás, Brazil; 3 Universidade do Estado do Rio de Janeiro, Rio de Janeiro, Brazil; 4 Instituto de Cardiologia de Porto Alegre, Porto Alegre, Brazil; 5 Serviço de Emergência em Cardiologia da Universidade de Pernambuco, Universidade de Pernambuco, Recife, Brazil; 6 Escola de Ciências Médicas de Minas Gerais, Fundação Educacional Lucas Machado, Belo Horizonte, Brazil; 7 Universidade Estadual de Ciências da Saúde de Alagoas, Maceió, Brazil; 8 Instituto Dante Pazzanese de Cardiologia, São Paulo, Brazil; 9 Universidade Federal de São Paulo, São Paulo, Brazil; 10 Duke University, Division of Cardiology, County Durham, United States of America; 11 Unidade de Hipertensão Arterial da Universidade Federal de Goiás, Brazil; 12 Abat Oliba CEU University, Barcelona, Spain

**Keywords:** Hypertension, blood pressure, population groups, ethnicity, Afro-descendants

## Abstract

**Background:**

The prevalence of hypertension (HT) and blood pressure (BP) control varies among ethnic-racial groups, but studies on this issue and correlations between BP and body mass index (BMI) in the black Brazilian population are scarce.

**Methods:**

Cross-sectional study in individuals included in the First Brazilian Hypertension Registry. Relationships between variables were analysed by a binary logistic regression analysis.

**Results:**

The study evaluated 2.191 (82.9%) non-Afro-descendant participants and 452 (17.1%) Afro-descendants. The median age was 61.9 years (55.3% women), the BMI was 28.4 kg/m² and the waist circumference (WC) was 93 cm in the former cohort. In the Afro-descendant group, the median age was 62.5 years (57.5% women), the was BMI 29.8 kg/m² and the was WC 98 cm. A significant correlation was identified between BMI and office diastolic BP (DBP) (*R* = 0.126; *p* = 0.007) in Afro-descendants. These individuals had 1.40 times the chance of being obese compared to those of other ethnicities (95% CI: 1.14–1.72; *p* < 0.001). Afro-descendant men had 0.78 times fewer chance of being obese compared to women (95% CI: 0.66–0.90; *p* = 0.002), and 1.49 times higher chance (95% CI = 1.21–1.82; *p* < 0.001) of having uncontrolled BP, with no differences with Afro-descendant women (HR 0.91; 95% CI = 0.78–1.07; *p* < 0.258).

**Conclusion:**

No correlations were found between office BP, BMI and WC, except for a very weak correlation between DBP and BMI in the Brazilian Afro-descendants, although they were 1.40 times more likely to be obese. In contrast, a significant correlation between SBP and BMI was observed in the non-Afro-descendants. Differences in blood pressure control were not identified between the sexes within each group, but only between ethnic groups, with people of African descent having a 1.49 times greater risk of uncontrolled hypertension compared to non-Afro-descendants.

## Introduction

Hypertension (HT) is one of the leading causes of cardiovascular diseases (CVD) and all-cause mortality in the world, with a greater impact in middle and low-income countries, despite the availability of safe, well-tolerated and accessible therapies (1). It is the most significant modifiable cardiovascular (CV) risk factor contributing to the high burden of CV morbidity and mortality (2). The prevalence of HT is increasing globally, with more than 1.3 billion people currently affected, representing one in every five adults (3). In addition to HT, obesity represents a substantial public health challenge and is an important risk factor for the onset and progression of atherosclerosis and its CV consequences (4).

Overweight and obesity have experienced a significant increase over time. Between 1980 and 2013, the proportion of individuals with a body mass index (BMI) ≥ 25 kg/m² rose from 28.8% to 36.9% in the male population and from 29.8% to 38.0% in the female population globally. In 2014, nearly half of the population of Brazil (52.4%) was overweight and 17.9% was obese (5,6,7).

Overweight and abdominal obesity are significant factors in triggering HT in the elderly. Conventional anthropometrical measures, such as BMI and waist circumference (WC), have been used as indicators of obesity and the risk of metabolic disorders. The risk of HT in both sexes increases significantly as WC increases (8). A significant rise in CV mortality associated with obesity has been observed in the US population over the past two decades (9).

Racial disparities in obesity raise questions about their underlying causes, highlighting the need to investigate the mechanisms behind these differences (10). Despite the widespread use of BMI in studies on obesity and vascular disease, the importance of WC has not been fully understood in this context (11). Although it is well known that different patterns of obesity have a significant impact on the risk of HT (12).

Some studies have shown a positive association between WC and arterial stiffness. This highlights the importance of assessing the impact of WC on vascular health as an additional approach for the early treatment of CVD and the prevention of adverse clinical outcomes (13,14). Furthermore, CV mortality related to obesity is increasing and shows significant variations according to ethnicity, sex and geographical location (9,15,16,17).

Due to the scarcity of data on BP control in the Afro-descendant Brazilian population, and also about the correlations between office BP in different ethnic-racial groups, this study aimed to investigate the association between self-declared skin colour and BP control, BMI and the correlations of office BP with anthropometric data in Afro-descendants and non-Afro-descendants Brazilians with hypertension.

## Methods

All data were obtained from the First Brazilian Hypertension Registry (1RBH) (18), a multicentre observational study approved by the Ethics Committee of the Hospital das Clínicas of the Federal University of Goiás, located in Goiânia, Brazil, on 17/02/2014, under protocol number 532146. The study included participants from all regions of Brazil, coming from public, private and mixed healthcare services. Data collection took place between June 2013 and October 2015.

The registry was conducted in strict compliance with national and international guidelines, including the Helsinki Declaration, CNS Resolution 196/96, and all its supplementary CNS/MS resolutions, the ICH Good Clinical Practice guidelines (1996), the Americas Document (2005) and Resolution 466/2012. Each clinical research centre submitted its study protocol. Informed Consent Forms (ICF), and all relevant documentation to the Research Ethics Committee (REC) of their institution for analysis and prior approval before performing any procedures covered by the registry. After informed consent, medical records were thoroughly examined, and participants were interviewed to complete an electronic clinical registry form (eCRF).

Individuals who signed the ICF with a minimum age of 18 years, with a confirmed diagnosis of HT exhibiting an SBP ≥140 mmHg and/or DBP ≥90 mmHg measured in a seated position following a strict protocol (19), or those using antihypertensive medications were included. Those who declared themselves white, brown and Asian were considered non-Afro-descendants, and those who declared themselves black were considered Afro-descendants.

Exclusion criteria included the presence of renal failure requiring dialysis treatment, hospitalization at the time of inclusion or in the past 30 days, hemodynamic instability with the need for vasoactive drugs in the past 30 days, heart failure of functional class III or IV, pregnancy and/or breastfeeding, severe liver disease, psychiatric conditions preventing protocol compliance, a history of stroke or acute myocardial infarction within the last 30 days before inclusion, severe diseases as assessed by the researcher and cancers with a prognosis of survival <1 year.

## Statistical Analysis

Continuous variables were described using mean, standard deviation (SD), or median (interquartile range), depending on the normality of the data tested by the Shapiro-Wilk test. Categorical variables were presented numerically, along with their percentages. The Spearman correlation was applied to all variables due to the absence of a normal distribution. The interpretation of correlation indices was as follows: 0.00 to 0.19 (very weak correlation), 0.20 to 0.39 (weak correlation), 0.40 to 0.69 (moderate correlation), 0.70 to 0.89 (strong correlation) and 0.90 to 1.00 (very strong correlation) (20). In the binary logistic regression analysis, a 95% confidence interval (CI) was used. In this context, the categories ‘non-Afro-descendant’ and ‘non-obese’ were considered as the reference factor (level 1) for the analysis of the association between self-declared skin colour and obesity (defined as BMI ≥30 kg/m²). In the regression analysis investigating controlled blood pressure, the ‘non-Afro-descendant’ population and the female sex were established as reference levels (level 1). Individuals with uncontrolled BP were defined as those with SBP ≥140 or DBP ≥90 or both (19). Jamovi software version 2.3 was used for statistical analysis.

## Results

A total of 2643 participants from 45 research centres across all regions of Brazil were included in the study, with 452 (17.1%) self-identifying as Afro-descendant. The study included 1234 (46.7%) patients attending public healthcare units, 822 (31.1%) in private units and 587 (22.2%) in mixed units. Afro-descendants and non-Afro-descendants participants were similar in terms of demographic characteristics, WC, hypertension treatment duration and the frequency of diabetes mellitus (DM). However, Afro-descendants had a higher BMI, shorter duration of DM and higher frequency of alcohol consumption compared to non-Afro-descendants (Table 1).

**Table 1:** Clinical characteristics of the cohort.


DESCRIPTION	*N*	NON-AFRO-DESCENDANT	AFRO-DESCENDANT	*p*

Self-declared skin colour	2643	2191 (82.9%)	452 (17.1%)	

Age, years	62.0 (54.1–69.4)	61.9 (54.1–69.4)	62.5 (54.2–69.7)	0.922

Female sex	1472 (55.6%)	1212 (55.3%)	260 (57.5%)	0.390

BMI, kg/m²	28.7 (25.6–32.0)	28.4 (25.5–31.9)	29.4 (26.0–32.9)	0.002

WC, cm	98.0 (90.0–107)	98.3 (90.0–107)	98.0 (91.0–106)	0.788

HT duration, years	10 (4–20)	10 (5–20)	10 (5–20)	0.044

HT treatment duration, years	10 (4–17)	10 (4–17)	10 (5–20)	0.072

DM	784 (29.66%)	656 (29.9%)	128 (28.3%)	0.492

DM duration, years	5.1 (3–14)	7 (3–15)	6 (2–10)	0.007

Smoking	165 (6.24%)	134 (6.1%)	31 (6.8%)	0.552

Alcohol consumption	197 (7.45%)	153 (6.9%)	44 (9.7%)	0.043


DM, diabetes mellitus; HT, hypertension; BMI, body mass index.

Correlations of office BP and anthropometric values in hypertensive non-Afro-descendant and Afro-descendant Brazilian population. In non-Afro-descendants, all correlations between BMI and WC with BP values were very weak (Table 2). Regarding the Afro-descendant participants, only a weak positive correlation between BMI and DBP was identified (Table 3).

**Table 2:** Correlations between office BP and anthropometric values in the hypertensive non-Afro-descendant Brazilian individuals (*n* = 2191).


VARIABLES		SBP	DBP

BMI	Rho	0.095	0.144

	*p*-value	<0.001	<0.001

WC	Rho	0.103	0.122

	*p*-value	<0.001	<0.001


The Spearman correlation was used for all variables. WC, waist circumference; BMI, body mass index; DBP, diastolic blood pressure; SBP, systolic blood pressure. Rho, 0.00–0.19 (very weak correlation); 0.20–0.39 (weak correlation); 0.40–0.69 (moderate correlation); 0.70–0.89 (strong correlation); 0.90–1.00 (very strong correlation).

**Table 3:** Correlations between office BP and anthropometric values in the hypertensive Afro-descendant Brazilian individuals (*n* = 452).


VARIABLES		SBP	DBP

BMI	Rho	0.09	0.126

	*p*-value	0.056	0.007

WC	Rho	0.106	0.051

	*p*-value	0.066	0.379


The Spearman correlation was used for all variables. WC, waist circumference; BMI, body mass index; DBP, diastolic blood pressure; SBP, systolic blood pressure. Rho: 0.00–0.19 (very weak correlation); 0.20–0.39 (weak correlation); 0.40–0.69 (moderate correlation); 0.70–0.89 (strong correlation); 0.90–1.00 (very strong correlation).

To investigate the association between ethnicity and obesity in hypertensive individuals, a logistic regression analysis was performed considering Afro-descendants and non-Afro-descendants Brazilians. Afro-descendant individuals had a 1.40 times higher chance of being obese compared to the non-Afro-descendants. Additionally, in both populations, men were less likely to be obese than women (Table 4).

**Table 4:** Binary logistic regression analysis: odds ratio (OR) and 95% confidence interval (CI) for obesity vs. non-obesity in hypertensive Afro-descendant (*n* = 452) and non-Afro-descendant Brazilians (*n* = 2191) stratified by sex.


INDEPENDENT VARIABLES	OR	CI (95%)	*p*

*Intercept*	0.67	0.60–0.75	<0.001

BMI			

Afro-descendant	1.40	1.14–1.72	<0.001

Non-Afro-descendant	1		

Sex (Afro-descendant)			0.002

Male	0.78	0.66–0.90	

Female	1		

Sex (Non-Afro-descendant)			0.005

Male	0.77	0.65–0.92	

Female	1		


Note: Estimates represent the log-odds of ‘BMI = Obese’ compared to ‘BMI = non-obese’ Obesity was defined as a BMI ≥30 kg/m².

Similarly, a logistic regression analysis was conducted to assess the blood pressure profile between the groups, with emphasis on the differences associated with ethnic self-declaration. Those who self-identified as black were 1.49 times more likely to have uncontrolled blood pressure compared to non-black individuals (Table 5).

**Table 5:** Binary logistic regression analysis for BP profile: uncontrolled vs. controlled BP among Afro-descendant and non-Afro-descendant Brazilian hypertensive individuals stratified by sex (*n* = 2643).


INDEPENDENT VARIABLES	OR	CI (95%)	*p*

*Intercept*	0.84	0.75–0.94	0.002

Self-declared skin colour			

Afro-descendants	1.49	1.21–1.82	<0.001

Non-Afro-descendants	1		

Sex			

Male	0.91	0.78–1.07	0.258

Female	1		


Note: Estimates represent the log-odds of ‘blood pressure profile = Uncontrolled’ compared to ‘Blood Pressure Profile = Controlled’. OR: Odds ratio; CI: confidence interval.

## Discussion

In contrast to the non-Afro-descendant population, Afro-descendants did not show any significant correlation between SBP and BMI or WC. Only the non-Afro-descendant population showed significant direct correlations between SBP and BMI. A higher likelihood of obesity was identified among Afro-descendant hypertensive individuals, as well as an increased risk of uncontrolled HT.

A previous Brazilian study revealed a positive correlation between BP and anthropometric measures, except for the waist-to-hip ratio, with WC being the only independent indicator related to HT (20). Another study involving more than 1000 individuals of all ethnicities found that both men and women had moderate correlations between height, WC, hip circumference, BMI and total body fat with SBP and DBP (21,22). General/abdominal obesity and HT showed a synergistic effect (23). Racial differences in body proportions – with Afro-descendants having a greater tendency towards mesomorphy and other anthropometric differences between ethnicities – may significantly affect BMI estimation and influence BP values differently (5,24,25).

Some studies indicate that the association between BMI and BP decreases over time (26,27), while others suggest that this relationship strengthens (28). These patterns seem to vary with age, suggesting a differential influence of factors over time (29). In a study analysing the relationship between obesity and BP in individuals aged 45 to 65 years without differentiation by ethnicity, SBP had a significantly moderate correlation with all obesity indices, in contrast to DBP. The obesity indices explained between 23.6% and 24.1% of the variability in SBP (30).

In a comprehensive study involving more than five million men in South Korea, the relationship between BMI and BP variability over time and across different population groups was investigated. The analysis revealed an annual increase in the correlation coefficient between BMI and SBP (from 0.257 to 0.495) and between BMI and DBP (from 0.164 to 0.413) (29). This indicated that as BMI increased, the annual rise in BP and BP per unit of BMI also increased.

A study conducted in the United Kingdom with more than 27,000 participants demonstrated that all demographic groups in the country are impacted by the increasing prevalence of HT, with a strong correlation between BMI and BP, both in the general population and in numerous subgroups (31).

In a study involving 928 adults without ethnic distinction and a mean age of 56 ± 10 years, a negative correlation was found between obesity indices, measured by BMI and WC, and the aortic augmentation index in both sexes, even after adjusting for mean BP (32).

In our study, no correlation was found between SBP and obesity indices (BMI or WC) in the Afro-descendant population, which differs from common results found in groups not stratified by ethnicity. This lack of correlation suggests that factors beyond weight and fat distribution (such as chronic stress, discrimination and socioeconomic inequalities) may have a more significant impact on BP regulation in Afro-descendants and may explain the observed differences.

It is worth noting that negative variations in income have also been linked to a higher risk of HT and lower BP control among Afro-descendants, underscoring the importance of socioeconomic status in CV health and suggesting that a dynamic approach to this status may be beneficial for understanding the factors influencing hypertension development (33,34). Our data showing that Afro-descendants in Brazil have 1.49 times more chance of having uncontrolled HT highlights the critical disparity in CV health.

The evidence that 34.5% of Afro-descendants live below the poverty line and that a significant portion occupies illegal housing points to an unfavourable socioeconomic context that may contribute to difficulties in managing hypertension (35). Also, disparities in perceptions about the risks of hypertension may affect control rates (36). This unfavourable scenario highlights that racial and income inequality in Brazil remains as deep as it is persistent. Both during the 2014–2016 crisis and the subsequent slow recovery, the per capita household income of Afro-descendant and mixed-race individuals continued to hover around 50% of the income of white individuals (37).

A study, carried out in the public health structure, with more than 14,000 participants in the United States investigating the social determinants of health in African American (AA) adults found that among participants using antihypertensive medications 25.4% of white individuals and 33.7% of AA individuals had uncontrolled BP. Deficient formal education, low purchasing power and residence in underserved neighbourhoods lacking basic urban services and healthcare professionals were associated with a higher likelihood of uncontrolled BP among AA adults compared to whites (38).

Higher-income families, generally, have greater awareness of the link between uncontrolled hypertension and renal disease, stroke and dementia compared to those with lower incomes. In an international study, non-Hispanic whites tended to be more aware of the health risks associated with poor habits than non-Hispanic African Americans, despite the latter having a higher prevalence of HT and uncontrolled HT (39).

The underlying causes of the higher prevalence and lower control rates of HT among ethnicities are very important points still discussed today, covering gene-environment interactions, work stress, racism and other psychosocial factors with ethnic disparities in the prevalence of HT (40,41,42).

Finally, our study has several limitations because the registry did not provide information on the underlying causes of social, educational, economic and behavioural disparities – such as access to healthcare services – treatment adherence or genetic factors which may limit the interpretation of the results. The inclusion of blood pressure measurements outside the office could have enriched the assessment of blood pressure control in the population studied.

## Conclusions

In conclusion, no significant associations were found between office BP and BMI or WC, except for a very weak correlation between DBP and BMI in the Brazilian Afro-descendant population, and direct correlations between SBP and BMI were only observed in the non-Afro-descendant population. Brazilian Afro-descendants had a higher likelihood of obesity and uncontrolled hypertension, without sex differences in the prevalence of uncontrolled hypertension.

## Data Accessibility Statement

The data from this study are not publicly available due to privacy restrictions and compliance with the Brazilian General Data Protection Law (LGPD). Access may only be granted upon formal request, ethical assessment and compliance with legal requirements. The data were obtained from the First Brazilian Hypertension Registry (1RBH), a multicentre observational study approved by the Research Ethics Committee of the Hospital das Clínicas of the Federal University of Goiás (protocol no. 532146). The study adhered to national and international guidelines, including the Declaration of Helsinki, CNS Resolution 466/2012 and the ICH Good Clinical Practice (1996). For more information, please contact the corresponding author.
